# The effect of TiO_2_ on nucleation and crystallization of a Li_2_O-Al_2_O_3_-SiO_2_ glass investigated by XANES and STEM

**DOI:** 10.1038/s41598-018-21227-x

**Published:** 2018-02-13

**Authors:** Enrico Kleebusch, Christian Patzig, Michael Krause, Yongfeng Hu, Thomas Höche, Christian Rüssel

**Affiliations:** 1 Otto Schott Institut, Jena University, Fraunhoferstraße 6, D-07743 Jena, Germany; 2 Fraunhofer Institute for Microstructure of Materials and Systems IMWS, Walter-Huelse-Straße 1, 06120 Halle (Saale), Germany; 3 Canadian Light Source Inc., University of Saskatchewan, 44 Innovation Boulevard, Saskatoon, S7N 2V3 Canada

## Abstract

Glass ceramics based on Li_2_O/Al_2_O_3_/SiO_2_ are of high economic importance, as they often show very low coefficients of thermal expansion. This enables a number of challenging applications, such as cooktop panels, furnace windows or telescope mirror blanks. Usually, the crystallization of the desired LAS crystal phases within the glasses must be tailored by a careful choice of crystallization schedule and type of nucleation agents to be used. The present work describes the formation of nanocrystalline TiO_2_ within an LAS base composition that contains solely TiO_2_ as nucleating agent. Using a combination of scanning transmission electron microscopy as well as X-ray absorption spectroscopy, it is found that a mixture of four- and six-fold coordinated Ti^4+^ ions exists already within the glass. Heating of the glass to 740 °C immediately changes this ratio towards a high content of six-fold coordinated Ti, which accumulates in liquid-liquid phase-separation droplets. During the course of thermal treatment, these droplets eventually evolve into nanocrystalline TiO_2_ precipitations, in which Ti^4+^ is six-fold coordinated. Thus, it is shown that the nucleation of nanocrystalline TiO_2_ is initiated by a gradual re-arrangement of the Ti ions in the amorphous, glassy matrix, from a four-fold towards a six-fold coordination.

## Introduction

Glass-ceramics based on the system Li_2_O/Al_2_O_3_/SiO_2_ (LAS) are very important for a wide range of applications^1–6^, due to their coefficients of thermal expansion which are often close to zero^1,7^. Although these materials are of high economic importance and widely used for more than 60 years, the fundamentals of the crystallization processes therein are still not fully understood^8^. A key issue for the manufacture of these glass-ceramics is the addition of nucleating agents^1,6,9–11^.

In previous studies we investigate different LAS glass compositions to verify the mechanism of nucleation especially in the early stages. In a composition which contains only ZrO_2_ as nucleation agent, phase separation droplets of ZrO_2_ could be proved^12^. The development of these droplets is accompanied by a change of the coordination state of zirconia from six to eight^13^. In compositions which contains both of the nucleating agents, ZrO_2_ and TiO_2_, these phase separation droplets are absent and ZrTiO_4_-crystals form the first step of nucleation^14^.

In order to study the effect of TiO_2_^1,11,15–19^ on the nucleation mechanism in a lithium aluminosilicate glass, a composition close to that of the commercially available ROBAX^TM^ glass (Schott AG), which, however, contains both, TiO_2_ and ZrO_2_^1,11,14,17,18,20,21^ as nucleating agents, was used in the present study. The addition of only one nucleating agent provides the possibility to discuss the mechanism of LAS crystallization in more detail and to point out the changes in the crystallization mechanism^12,19^. According to the model of microstructure evolution in LAS glass-ceramics as suggested in ref.^22^, early stages of crystallization were investigated in a former study^19^ if the composition contains only TiO_2_ as nucleating agent. In these early stages of crystallization, phase separation droplets were found^19,23^. Additionally, the development of these droplets with different thermal treatment times and temperatures was studied. It is found that nanocrystals of the nucleating agents precipitate at lower temperatures before the lithiumalumosilicate (LAS) phase is formed^12,19,23^. The LAS phase with a high-quartz structure crystallizes during thermal treatment at temperatures above T_g_^12,19^. These crystals are notably larger than the TiO_2_ crystals and they form a network that is growing equally with treatment time and temperature^12,19^.

In a recent study, we gave detailed insights into the course of crystallization in LAS glass ceramics with TiO_2_ as the only nucleating agent^19^. In that study, it could be already shown that if an LAS glass of the composition as given in Table 1 is thermally treated at temperatures *T* above ≈ 700 °C, for a certain period of time *t*, bulk crystallization occurs. Using XRD and analytical TEM techniques, it was proved that the glass sample already shows some tiny inhomogeneities, which can be interpreted as first signs of liquid-liquid phase separation within the glass matrix. When the glass is heated, the nanoscaled phase separation of droplets quickly become more apparent. From those droplets, upon further thermally treatment, TiO_2_ nanocrystals with diameters of less than 10 nm precipitate. With ongoing time, the LAS phase starts to crystallize upon these TiO_2_ nuclei, finally forming LAS crystals with diameters in the range of approximately 20 to 40 nm^19^.

**Table 1:** Chemical composition of the studied glass in mol%.

	Li_2_O	Na_2_O	K_2_O	MgO	BaO	ZnO	Al_2_O_3_	SiO_2_	TiO_2_	Sb_2_O_3_
glass	7.6	0.2	0.1	1.9	0.3	1.2	12.7	70.6	5.0	0.4

This sequence of crystallization events that take place in the TiO_2_-nucleated LAS glass was elaborately described via a careful analysis of the micro- nanostructural changes within the glass at several time steps *t* in the previous study^19^. In the present study, we go one step further, and complement these results with X-ray absorption spectroscopy (XAS) data to show that the crystallization of the TiO_2_ nanocrystals, in whose crystal lattices Ti ions are 6-fold coordinated by oxygen atoms, is initialized by a change of the Ti coordination from the glass. In the following, the coordination state of the nucleating agent itself during the crystallization process within an LAS glass is described. We investigate the temporal course of precipitation of TiO_2_ nanocrystals within a glassy matrix, at a given temperature of thermal treatment for several periods of time.

According to ref.^23^, it is possible to determine and describe the crystallization mechanism of the nucleation agent by using a combination of X-ray absorption near edge structure spectroscopy (XANES), XRD and (S)TEM. Here, the evolution of nanoscaled, Ti-rich liquid-liquid phase separation droplets into nanoscaled TiO_2_ crystals can be followed by using of this combination.

## Experimental

In a platinum/rhodium crucible, the glass was melted in batches of 300 g. The used raw materials were: Al(OH)_3_ (Sumitomo Chemical), BaCO_3_ (SABED), Li_2_CO_3_ (UCB), LiNO_3_ (Honeywell Riedel de Haën AG), 4 MgCO_3_·Mg(OH)_2_·4H_2_O (Merck KGaA), TiO_2_ (Germed DDR), Sb_2_O_3_ (Ferak Berlin GmbH), ZnO (Vertriebsgemeinschaft für Harzer Zinkoxide GmbH (VHZ), Heubach), ZrO_2_ (Tosoh), K_2_CO_3_, Na_2_CO_3_ and SiO_2_ (Carl Roth GmbH & Co. KG). In Table 1, the detailed composition is summarized.

The composition is related to a commercially available low thermal expansion glass ceramic, Robax™ glass (SCHOTT AG), with the exception that this glass ceramic contains both, ZrO_2_ and TiO_2_ as nucleating agent, while in the present glass only 5.0 mol% TiO_2_ occurs. The melting process is separated into two single steps in which the first is conducted in a middle frequency furnace for 2 h at a temperature of 1615 °C. The second step is carried out in a superkanthal furnace with MoSi_2_ heating elements to enable higher melting temperatures up to 1680 °C for additionally 3 h. The first step of this sequence allows the stepwise filling of the raw materials into the crucible subsequently followed by the second step to reach the necessary high melting temperature of 1680 °C. For casting of the glass melt, a brass mould was used. Then, the glasses were transferred into preheated muffle furnaces (660–680 °C). After switching off the furnace, the glasses slowly cooled down to room temperature with a cooling rate approximately between 2–3 K/min).

In order to study the temporal evolution of the TiO_2_ crystallization, the glass was cut into 0.5 × 0.5 × 0.5 cm^3^ pieces. These samples are given in a muffle furnace (Nabertherm) pre-heated to 740 °C for different times *t*, between *t* = 15 min to *t* = 24 h.

In order to perform XRD, the bulk samples were studied using a Rigaku MiniFlex300X -Ray Diffractometer with Cu-*K*_α_ radiation (λ ≈ 0.154 nm) in a 2θ-range from 10 to 60°.

The glass transition temperature and the coefficient of thermal expansion was determined by dilatometry using a Netzsch Dil 402-PC dilatometer, and specimens with a length of 25 mm and a diameter of 8 mm; the heating rate was 10 K/min. Differential scanning calorimetry was performed using a Linseis DSC Pt-1600 and a heating rate of 10 K/min. The density was measured with a helium pycnometer (AccuPyc 1330).

TEM sample preparation was performed via a purely mechanical approach: using a dedicated polishing and grinding tool (Allied company), the samples were plane-parallel polished to a residual thickness of approximately 20 µm. Then, further polishing was done under a defined, very low angle (1.6°), until a very thin, electron transparent wedge was prepared at one sample side. To clean the sample from polishing residues and to finally reduce the wedges thickness even further, the wedge-polishing procedure was followed by a glancing angle Ar^+^ ion beam milling step.

X-ray absorption near-edge structure (XANES)-spectroscopy at the Ti *K*-edge was performed at the Canadian Light Source (CLS) in Saskatoon, SK, Canada. The experiments were run at the soft X-ray micro characterization beamline (SXRMB). As monochromator, Si single crystals, cut in (111) direction, were used, establishing a resolving power of 10^4^. The samples were fixed to the sample holder with double-sided conducting carbon tape. The experiments were run in vacuum, with a residual pressure of approximately 10^−8^ mbar. The fluorescence yield (FY) data were recorded with an Si-Li drift detector. For the Ti *K*-edge, an energy range between 4,915 and 5,080 eV was studied. The FY data were normalized by setting the averaged post-edge crest energy (greater 5,050 eV) to “1” and the pre-edge energy to “0”.

The micro- and nanostructure of selected samples was further studied using scanning transmission electron microscopy (STEM), with a *c*_*s*_-abberation corrected FEI TITAN^3^ 80–300 electron microscope using an acceleration voltage of 80 kV. The microscope is equipped with a high-angle annular dark field (HAADF) detector (Fischione Model 3000) to perform scanning TEM.

## Results and Discussion

The casted glass has a brown coloration frequently denoted as ilmenite coloration which is due to an Fe^3+^-O-Ti^4+^ charge transfer formed by trace impurities of iron^24–27^. The chemical composition was controlled by EDXS spectroscopy and agreed well with that calculated from the batch composition. It should be noted that Ti^3+^ does not occur in the glass in noticeable quantities. This would lead to a dark coloration, since the extinction coefficients of Ti^3+^ are very high. Also in the crystallized samples, a dark coloration is not observed. Hence, noticeable concentrations of Ti^3+^ do not occur, neither in the glass nor in the glass ceramic. As already described in ref.^19^ T_g_ of the studied glass determined by DSC is 663 °C, while the determination by dilatometry resulted in a value of 666 °C. The CTE of the glass was 4.04 × 10^–6^ K^−1^ in the temperature range from 100 to 500 °C and the density of the glass is 2.47 g/cm^3^.

The focus of this study is the temporal evolution concerning the nucleation agent TiO_2_ rather than on the subsequent LAS phase formation itself. For this purpose, numerous glass samples were thermally treated at 740 °C for different periods of time between *t* = 15 min and *t* = 24 h. Previous XRD studies in glasses with ZrO_2_ as nucleating agent showed that, ZrO_2_ precipitates during thermal treatment at 720 °C for 24 h, however, LAS does not yet precipitate at this temperature^12,13^. Otherwise, crystallization at 730 °C for 24 h, already leads to LAS crystallization as indicated by XRD^12^. The supplied temperature of 740 °C was chosen to initiate nucleation, however, without subsequent crystallization of the LAS phase. This should enable to study the nucleation agent within the LAS glass alone^13^. Otherwise, the nucleation step could potentially be affected by LAS crystallization.

Previous studies are related to LAS glass ceramics with ZrO_2_ as nucleation agent, in which it was proven that the crystallization especially of the nanocrystalline ZrO_2_-phase is accompanied by the formation of a nucleation barrier (core-shell structure) that prohibits classical crystal growth via Ostwald ripening. The scope of this study was to analyze whether the same, non-classical crystal growth effect does also take place in a glass, which contains TiO_2_ as a different type of nucleation agent.

### XRD

In the following, a brief summary of the XRD results^19^ is given.

**Figure 1:**
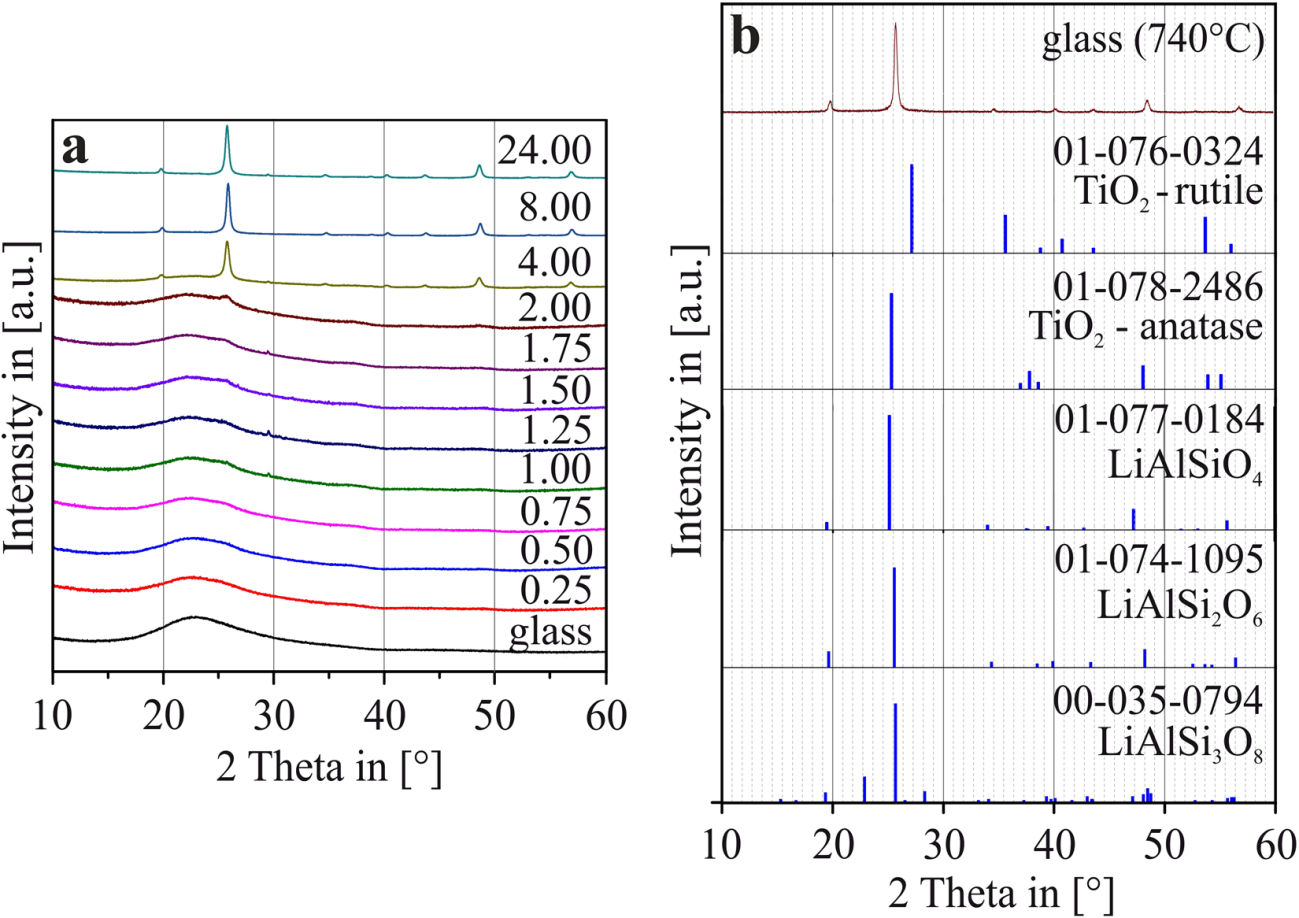
(**a**) Series of XRD θ−2θ-patterns of the LAS glass sample, thermally treated at 740 °C for several thermal treatment times *t*. (**b**) XRD θ-2θ-patterns of the LAS glass sample, thermally treated at 740 °C for *t* = 24 h, and theoretical JCPDS patterns of the possible phases that can be expected to crystallize within the glass.

Figure 1(a) shows an XRD θ−2θ-scan of a time series of the TiO_2_-nucleated glass samples with a composition as given in Table 1, all thermally treated at the same temperature of *T* = 740 °C. At short treatment times *t*, up to approximately *t* = 1 h, no indications on crystalline phases are obtained. The observed broad diffraction hump between approximately 2θ = 20 and 25° is a typical signal found in any amorphous material (although the respective 2θ-range depends on glass composition)^14^. After thermal treatment for *t* = 1 h, indications of a peak at 2θ ≈ 25.1° are seen^19^, as well as slight indications of a peak at 2θ ≈ 48.1°. These peaks are an indication for crystalline TiO_2_ in anatase configuration (JCPDS no. 01–078–2486), in which Ti is present in 6-fold coordination (^[6]^Ti). The XRD pattern alone do not enable an unambiguous assignment to TiO_2_- or LAS-phases. The evidence for the appearance of TiO_2_ in anatase modification is given in ref.^19^ by a combination of XRD results with analytical (S)TEM and energy-dispersive X-Ray spectroscopy (EDXS). After *t* = 4 h, these TiO_2_ - related peaks become very distinct, and additionally, further peaks can be discerned at 2θ ≈ 19.8/28.3/34.7/40.2/43.7/48.5/56.9°, which are not linked to TiO_2_. These low intensity peaks are also due to the occurrence of a type of β-quartz and can be attributed to either of the LAS phases LiAlSiO_4_ (JCPDS no. 01–077–0184), LiAlSi_2_O_6_ (JCPDS no. 01–074–1095) or LiAlSi_3_O_8_ (JCPDS no. 00–035–0794) that apparently crystallizes as second phase from this glass during thermal treatment at *T* = 740 °C^12,19,21,28^. Figure 1(b) XRD θ−2θ-patterns of the LAS glass sample, thermally treated at 740 °C for *t* = 24 h is compared to the theoretical JCPDS patterns of the possible phases that can be expected to crystallize within the glass.

The XRD results as presented above allow general assumptions concerning the course of crystallization upon thermal treatment of the LAS glass with solely TiO_2_ as nucleation agent^19^: starting from an amorphous glass sample, at first, TiO_2_ is precipitated, followed by a crystallization of the LAS phase later on.

Nevertheless, these results give no insight into (possible) changes of the coordination of the Ti ions within the glass during the crystallization process, and furthermore, details concerning the crystal sizes, crystal density, and the developing micro- and nanostructure during formation of the glass ceramics cannot be derived. Furthermore, information concerning the appearance of amorphous liquid-liquid phase separations cannot be deduced from the XRD data. In order to give detailed insights into these points, in the following, XAS and also some further analytical TEM results will be presented, to shed further light onto the processes that are involved during crystallization of this multicomponent LAS glass.

### XAS and STEM

**Figure 2:**
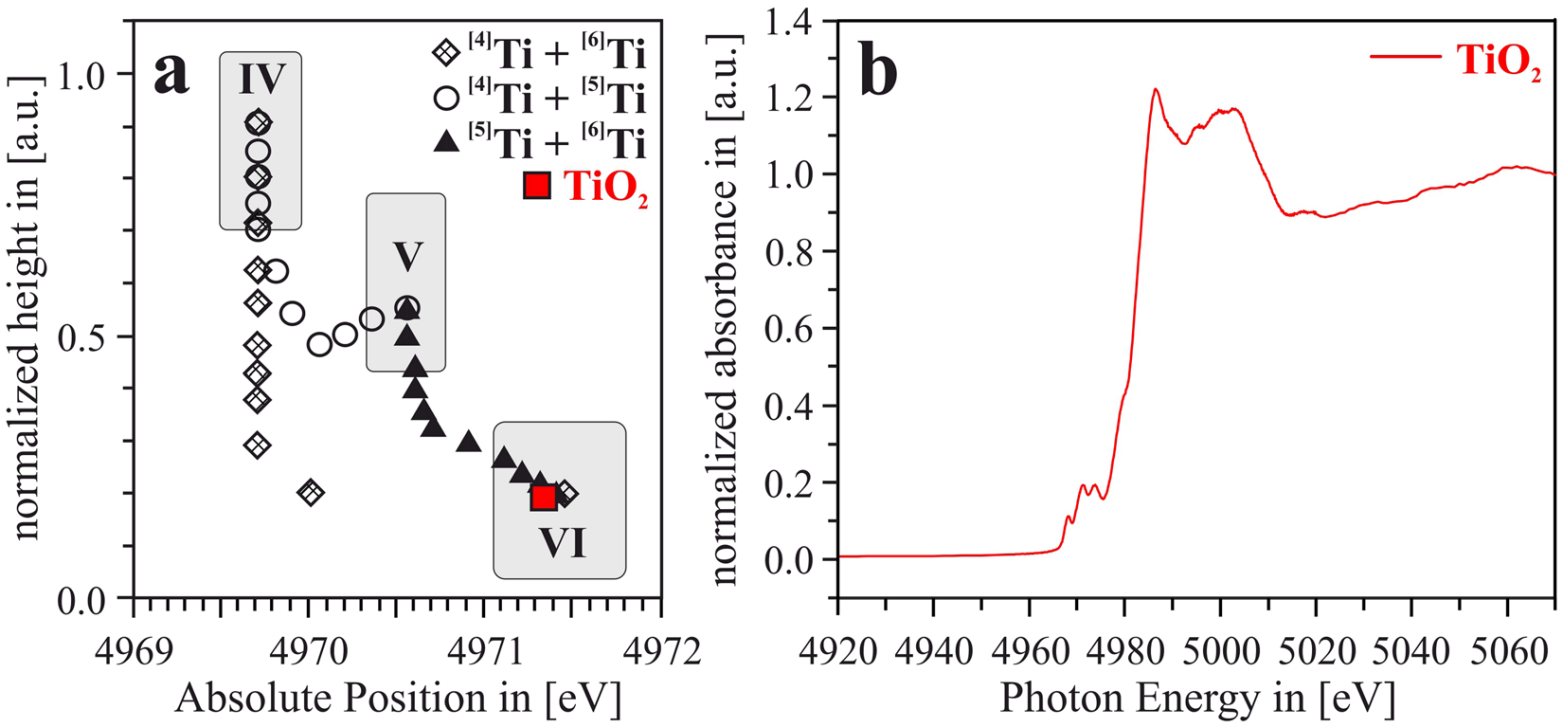
(**a**) Representation of normalized height vs. absolute energetic position of the Ti-K pre-peak. The grey areas indicate which combination of absolute position and normalized height is indicative for ^[4]^Ti, ^[5]^Ti and ^[6]^Ti. [redrawn from ref.^29^]. The triangles, squares and diamonds show predicted normalized height vs. absolute position – values for compositions of ^[4]^Ti + ^[6]^Ti, ^[4]^Ti + ^[5]^Ti, ^[5]^Ti + ^[6]^Ti with different ratios of the respective composition [see ref.^29^]. The analyzed TiO_2_ powder standard is apparently 6-fold coordinated, as expected. (**b**) Spectrum of the TiO_2_ powder reference, the average absorption coefficient for energies greater than 5050 eV is set as “1”, and the spectral data is normalized to that value.

According to Farges *et al*., who ran extensive studies concerning the determination of the coordination of Ti in crystalline and glassy compounds^29–33^, the energetic position of the Ti-K pre-peak feature, as well as its normalized height with respect to the post-edge crest, are very sensitive to the coordination of the Ti ions within the samples. By XAS analyses of a large number of crystalline compounds with Ti in either four-, five-, or six-fold coordination, it was shown that the Ti-K pre-edge peaks of ^[4]^Ti compounds are positioned at around 4969.5 eV with normalized heights of ≈0.8–1.0^29–31^, whereas those of ^[5]^Ti compounds and ^[6]^Ti compounds are positioned at higher energies and exhibit lower normalized heights, see Fig. 2(a)^29–31^. Moreover, according to^29^, it is possible to predict the absolute position and normalized height of the Ti-K pre-peak of compounds with mixed Ti coordination states, see Fig. 2(b).

At first, the energy calibration of the beamline was checked by running an XAS scan at the Ti K-edge of the TiO_2_ anatase powder standard^29^. The scan is shown in Fig. 2(b), and the normalized height vs. absolute position of the Ti-K pre-peak is also shown in Fig. 2(a) – positioned well in the expected range for a ^[6]^Ti compound such as TiO_2_. Thus, it can be assumed that the energy calibration of the beamline is correct, and a direct comparison of the Ti K-edge results gained in this study with the literature data reported by Farges *et al*. seams feasible^29^.

**Figure 3:**
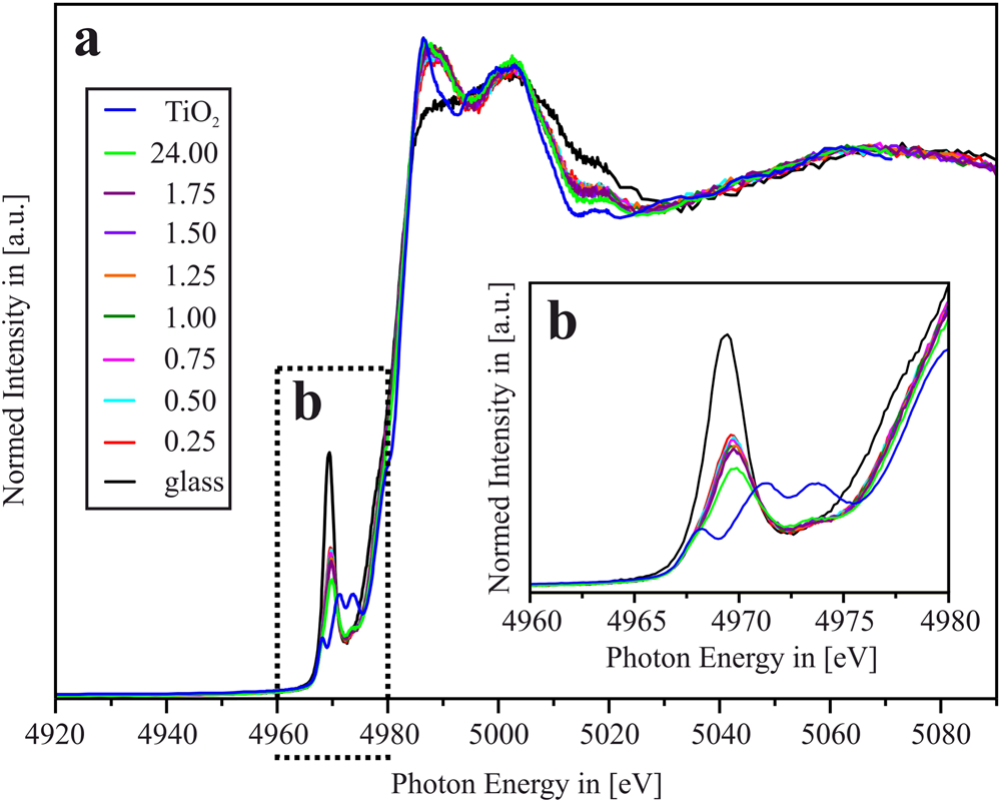
(**a**) X-Ray absorption spectra at the Ti K-edge of samples that were thermally treated at 740 °C for various times between *t* = 0.25–24.00 h, in comparison to the spectrum of TiO_2_. The inset (**b**) shows that the relative position of the pre-peak shifts to higher energies with increasing time of thermal treatment, whereas the normalized height decreases with increasing treatment time.

Figure 3 shows the Ti K-edge XAS of the glass and the samples thermally treated at 740 °C for different treatment times *t* between *t* = 15 min and *t* = 24 h. Obviously, the sheer thermal treatment process itself leads to a drastic reduction of the normalized height of the Ti-K pre-peak, as well as to a slight shift of the energetic position of the latter towards higher energies. With increasing thermal treatment time, this trend continues in a moderate way, until finally, at *t* = 24 h, the lowest value of normalized pre-peak height and highest absolute energetic value of the latter is reached.

**Figure 4:**
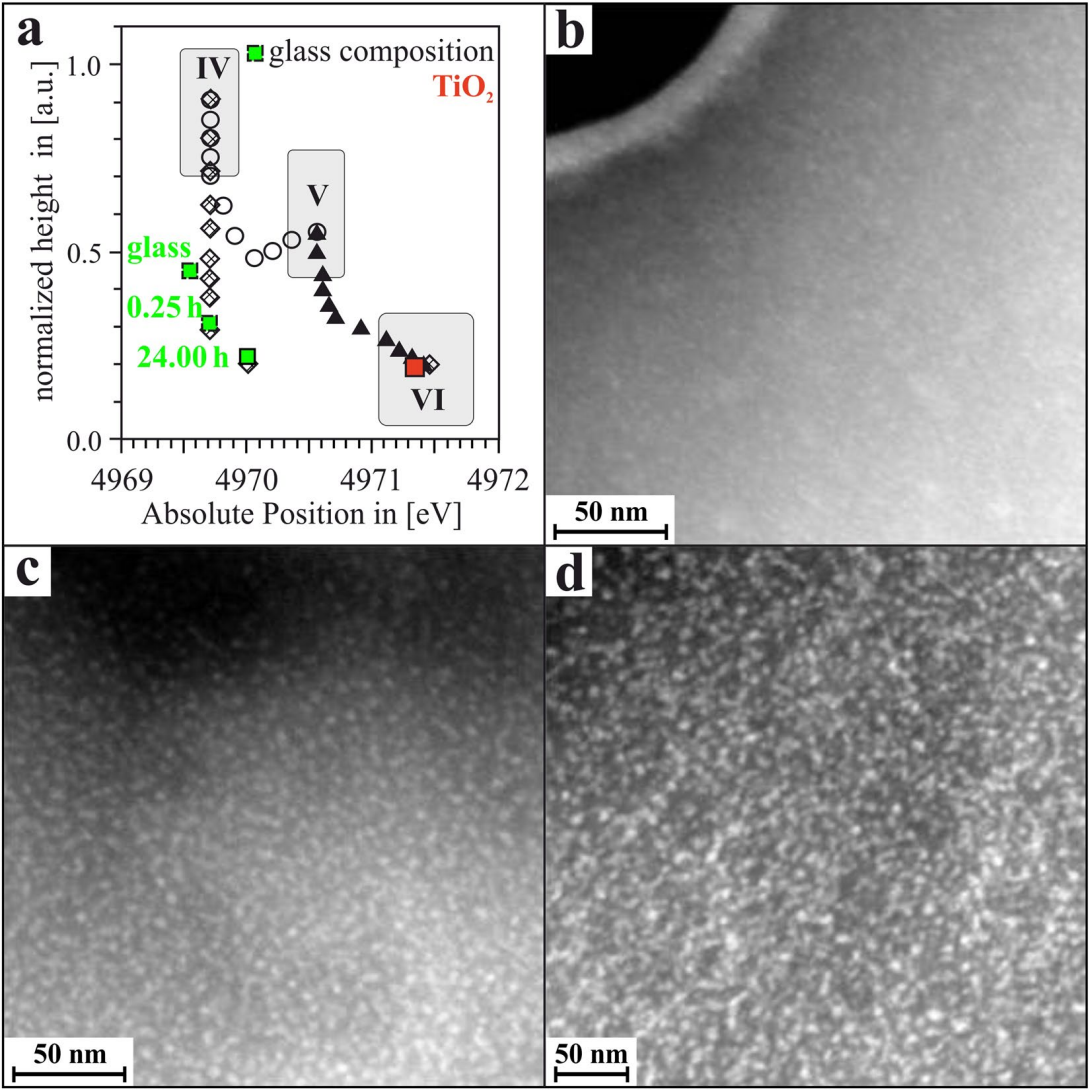
(**a**) Normalized height vs. absolute energetic position of the Ti-K pre-peak of the glass sample and the samples that were thermally treated at 740 °C for *t* = 15 min and *t* = 24.00 h redrawn from Farges *et al*. [see ref.^29^]. STEM-HAADF images of (**b**) the LAS glass sample, (**c**) the *t* = 15 min sample and (**d**) the *t* = 24.00 h sample.

If these values are compared with the data presented by Farges *et al.*^29^ as shown in Fig. 2(b), it is obvious that already in the glass, the Ti ions are not solely four-, five- or six-fold coordinated. Rather, the amorphous matrix contains a mixture of ^[4]^Ti and ^[6]^Ti, which should be roughly ^[4]^Ti:^[6]^Ti ≈ 50:50, as shown in Fig. 4(a). Heating up the glass to 740 °C leads to a drastic change of the ^[4]^Ti:^[6]^Ti ratio. Even after very short treatment times (*t* = 15 min), at which, according to XRD (cf. Fig. 1) as well as to TEM results (as presented in^19^ and further on in this manuscript), a precipitation TiO_2_ did not start yet, the ratio of four-fold- to six-fold-coordinated Ti ions within the still amorphous matrix is now ^[4]^Ti:^[6]^Ti ≈ 20:80. With increasing times of thermal treatment, this ratio gradually shifts to approximately ^[4]^Ti:^[6]^Ti ≈ 10:90 for the *t* = 24 h – sample.

TEM micrographs as well as selected area electron diffraction performed with glass samples did not give any hint at the occurrence of crystals. These results show that even in the glass itself, apparently, first re-arrangements of zones with different Ti coordination within the overall amorphous matrix must have formed. Indeed, as the STEM-HAADF micrograph in Fig. 4(b) shows, the glass itself is not totally homogeneous on the nanoscale: numerous, very small inclusions, with diameters in the range of ≈2 to 3 nm, are visible. After *t* = 15 min, as the STEM-HAADF micrograph in Fig. 4(c) shows, this microstructure is more prominent, with a higher density of slightly larger inclusions within the glassy matrix as compared to the glass sample itself. As exemplified in the previous study on this topic^19^, at *t* = 15 min, these inclusions are enriched in Ti. On the other hand, the whole sample is still amorphous, indicating that these inclusions are non-crystalline, liquid-liquid phase separation droplets. The abrupt change of the ^[4]^Ti:^[6]^Ti ratio from ≈50:50 (in the glass sample) to ≈20:80 (after *t* = 15 min) shows that drastic re-arrangements of the Ti ions and their respective coordination take place within the glass during the thermal treatment process. Most likely driven by temperature-induced diffusion, Ti ions from the glass matrix diffuse to the once tiny (≈2–3 nm) liquid-liquid phase separation droplets which during the course of this process form larger (≈5–10 nm) droplets. Within those, Ti arranges as ^[6]^Ti as a pre-stage to the crystallization of TiO_2_ nanocrystals, which, according to XRD and TEM data, does not take place before *t* ≈2 h. The fact that the most drastic change of the ^[4]^Ti:^[6]^Ti ratio already takes place during thermal treatment of only *t* = 15 min. Afterwards, the further increase of ^[6]^Ti is only gradual and shows that the crystallization of TiO_2_ from these glasses is not a sudden process that instantaneously changes the coordination of Ti from (mostly) four-fold (in the glass) to six-fold (in TiO_2_). The gradual change of the Ti coordination from ^[4]^Ti to ^[6]^Ti during thermal treatment takes place in a still-amorphous matrix via liquid-liquid phase separation and later eventually leads to the crystallization of TiO_2_ nanocrystals, that are shown in Fig. 4(d).

Nevertheless, although it is assumed that the “overall” TiO_2_ crystallization in the LAS glass is completed after *t* ≈ 24 h, as the LAS phase has already started to precipitate as secondary phase. Then, from the XAS data, it is obvious that not all Ti of the sample has been transformed into TiO_2_. The Ti K-edge spectrum of the *t* = 24 h sample is still different from that of the TiO_2_ reference spectrum (see Fig. 3), and, according to Fig. 4(a), it can still be assumed that approximately 10% of the Ti within the *t* = 24 h sample is still present in four-fold coordination. Hence, it can be concluded that not all Ti ions have been incorporated into the TiO_2_ nanocrystals, yet a fraction of the Ti is still present within the residual glass matrix in which the TiO_2_ and LAS crystals are embedded.

**Figure 5:**
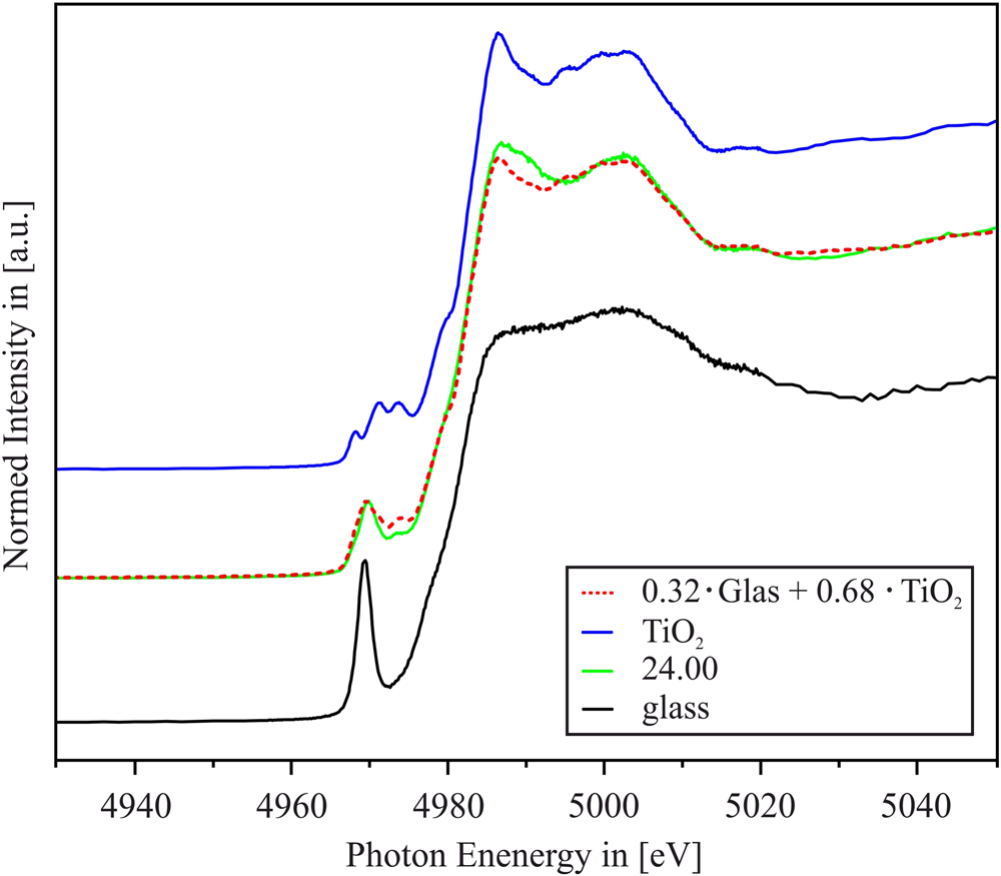
Linear combination (dashed line) of 32% of the green glass Ti K-edge XANES spectrum (bottom) and 68% of the TiO_2_ reference Ti K-edge spectrum (top) in comparison to that of the *t* = 24 h sample.

If it is assumed that the residual glass matrix after *t* = 24 h comprises Ti ions in both six-fold and four-fold coordinations, just as it is the case for the initial glass sample without any temperature treatment, it should be feasible to represent the XAS Ti K-edge spectrum of the *t* = 24 h sample by a linear combination of the Ti K-edge XAS spectra of the initial glass (representing the fraction of Ti within the residual glass that contributes to the spectrum of the *t* = 24 h sample), and the Ti K-edge XAS spectra of the TiO_2_ reference (representing the fraction of Ti within the TiO_2_ nanocrystals that contributes to the spectrum of the *t* = 24 h sample). In Fig. 5, it is shown that the best fit can be achieved – in a qualitative way - if the *t* = 24 h - spectrum is compared to a linear combination of a weighted fraction of 32% of the green glass spectrum, and 68% of the TiO_2_ spectrum. If it is assumed that 50% of the Ti ions within the residual glass are six-fold coordinated (just as it is the case in the initial glass), this means that the half of this 32% - fraction, i.e., 16% of the total Ti ions within the sample, consists of six-fold coordinated Ti ions incorporated in the residual glass. Together with 68% of ^[6]^Ti within the TiO_2_ nanocrystals, the amount of ^[6]^Ti within the *t* = 24 h sample should be roughly 84% - which is close to the previous finding that the ^[4]^Ti:^[6]^Ti ratio should be approximately 1:9, as shown in Fig. 4(a). The best fit of the *t* = 24 h – spectrum shows deviations to the actual spectrum, especially in the energetic range directly behind the Ti K-edge whiteline. This might be due to the fact that the spectrum of the initial glass, used as one reference spectrum for the linear combination, is not the perfect choice because it is well possible that the ratio of ^[4]^Ti:^[6]^Ti in the initial glass is different from that of the residual glass after thermal treatment. As already pointed out, the green glass is not totally homogeneous, but shows already some phase separation. Hence, it might be that the ratio of ^[4]^Ti:^[6]^Ti ≈ 1:1 within the initial glass sample is attributed to the glass itself, which might contain only ^[4]^Ti ions, and tiny, liquid-liquid phase separation droplets, which might well contain all ^[6]^Ti ions that are present in the initial glass sample. In the later stages, the TiO_2_ nanocrystals start to crystallize from these phase separation droplets.

## Conclusion

The present investigation reports on the crystallization behavior of a glass with a chemical composition comparable to that of the commercially available glass-ceramic material Robax™. It was, however, deliberately doped with TiO_2_ as the only nucleating agent, in order to analyze the crystallization behaviour of this nucleating agent before a subsequent LAS crystallisation.

It was shown that the crystallization of TiO_2_ is initiated by a successive change of the Ti coordination within the still amorphous glassy matrix. Starting from a glass with mixed coordination states (^[4]^Ti:^[6]^Ti ≈ 50:50), the amount of ^[6]^Ti within the glass already increases if it is heated up to 740 °C and held for short times (*t* = 15 min) only. At the same time, liquid-liquid phase separation droplets enriched in Ti^4+^ form in the still-amorphous samples. With ongoing time of thermal treatment, crystalline TiO_2_ starts to precipitate, a process initialized by a re-arrangement of the coordination of the Ti ions in the glass matrix from a mixture of four- and six-fold coordination to mainly six-fold coordinated Ti ions, which is a prerequisite to the formation of TiO_2_ (with Ti in six-fold coordination). These TiO_2_ nanocrystals act as nuclei for a subsequent precipitation of the LAS crystal phases at the later stages of thermal treatment. Nevertheless, even after *t* = 24 h, when LAS crystals already exist in the samples, there is still a small amount of four-fold coordinated Ti ions present in the samples, apparently in the residual glass matrix. Most likely, the re-arrangement of the Ti coordination from four-fold to six-fold within the glass, and the precipitation of the LAS phase (that evolves as a result of the existence of nanocrystalline TiO_2_, which had already formed earlier in the crystallization process) are running parallel and somewhat concurring after a certain treatment time.

The precipitation of the TiO_2_ nanocrystals, is accompanied by a change of the Ti coordination from the glass in which a mixture of four- and six-fold-coordinated Ti ions occurs to an ever-increasing ^[6]^Ti concentration within the samples with ongoing thermal treatment time.

Once LAS starts to form, the residual glass is spatially more and more separated, making it more and more unlikely for the still four-fold coordinated Ti ions therein to diffuse to the TiO_2_ crystals or the not-yet crystallized Ti-rich phase-separation droplets.
